# Do Sustained Lung Inflations during Neonatal Resuscitation Affect Cerebral Blood Volume in Preterm Infants? A Randomized Controlled Pilot Study

**DOI:** 10.1371/journal.pone.0138964

**Published:** 2015-09-25

**Authors:** Bernhard Schwaberger, Gerhard Pichler, Alexander Avian, Corinna Binder-Heschl, Nariae Baik, Berndt Urlesberger

**Affiliations:** 1 Research Unit for Cerebral Development and Oximetry, Medical University of Graz, Graz, Austria; 2 Research Unit for Neonatal Micro- and Macrocirculation, Medical University of Graz, Graz, Austria; 3 Division of Neonatology, Department of Pediatrics, Medical University of Graz, Graz, Austria; 4 Institute for Medical Informatics, Statistics and Documentation, Medical University of Graz, Graz, Austria; Centre Hospitalier Universitaire Vaudois, FRANCE

## Abstract

**Background:**

Sustained lung inflations (SLI) during neonatal resuscitation may promote alveolar recruitment in preterm infants. While most of the studies focus on respiratory outcome, the impact of SLI on the brain hasn’t been investigated yet.

**Objective:**

Do SLI affect cerebral blood volume (CBV) in preterm infants?

**Methods:**

Preterm infants of gestation 28 weeks 0 days to 33 weeks 6 days with requirement for respiratory support (RS) were included in this randomized controlled pilot trial. Within the first 15 minutes after birth near-infrared spectroscopy (NIRS) measurements using ‘NIRO-200-NX’ (Hamamatsu, Japan) were performed to evaluate changes in CBV and cerebral tissue oxygenation. Two groups were compared based on RS: In SLI group RS was given by applying 1–3 SLI (30 cmH_2_O for 15 s) continued by respiratory standard care. Control group received respiratory standard care only.

**Results:**

40 infants (20 in each group) with mean gestational age of 32 weeks one day (±2 days) and birth weight of 1707 (±470) g were included. In the control group ΔCBV was significantly decreasing, whereas in SLI group ΔCBV showed similar values during the whole period of 15 minutes. Comparing both groups within the first 15 minutes ΔCBV showed a tendency toward different overall courses (p = 0.051).

**Conclusion:**

This is the first study demonstrating an impact of SLI on CBV. Further studies are warranted including reconfirmation of the present findings in infants with lower gestational age. Future investigations on SLI should not only focus on respiratory outcome but also on the consequences on the developing brain.

**Trial Registration:**

German Clinical Trials Register DRKS00005161 https://drks-neu.uniklinik-freiburg.de/drks_web/setLocale_EN.do

## Introduction

Transition from fetal to extra-uterine life is dependent upon physiological changes in the cardiovascular and respiratory system at birth. During postnatal transition many preterm infants need respiratory support to assist lung aeration and establish a functional residual capacity (FRC), which seems to be the key to successful neonatal resuscitation. Initial ventilatory strategies may play an important role in the development or prevention of lung and brain injury [1–6].

The approach of applying sustained lung inflations (SLI) in the delivery room is of growing interest, but remains controversial. While some guidelines recommend SLI to be considered in infants showing insufficient breathing [7], others address concerns about the use of SLI due to lack of evidence [8].

Animal studies showed that SLI immediately after birth may support transition to breathing by promoting lung aeration and alveolar recruitment [9, 10]. A prolonged 20 second SLI at birth was capable to recruit a FRC but not to decrease lung injury from continued mechanical ventilation [11]. To avoid lung collapse at the end of expiration and the development of atelectotrauma, the effect of positive end-expiratory pressure (PEEP) is crucial. Both, SLI and PEEP seem to have synergistic effects on achieving and maintaining a FRC and a rapid initiation of effective pulmonary gas exchange [9].

In some clinical studies benefits of SLI in regard to respiratory outcome were shown. *Lista et al*. reported that SLI and consecutive nasal continuous positive airway pressure (CPAP) treatment reduced the need for mechanical ventilation in the first 72 hours compared with CPAP-only treatment, but did not reduce the incidence of bronchopulmonary dysplasia [12]. *Te Pas et al*. found a decrease in need for mechanical ventilation at 72 hours after birth and a lower incidence of bronchopulmonary dysplasia in preterm infants treated with repeatable SLI maneuvers in the delivery room compared to standard treatment [4]. In contrast, *Lindner et al*. didn’t find beneficial effects on respiratory outcome in preterm infants initially treated with SLI in the delivery room [13]. Recently, a meta-analysis showed a decrease in requirement for mechanical ventilation within 72 hours of life after SLI treatment but no improvement in the rate of bronchopulmonary dysplasia and/or death [14].

While most of the studies focused on respiratory outcome, the impact of SLI on the developing brain has not been examined yet. By using a high inspiratory pressure for many seconds, SLI may increase intra-thoracic pressure and therefore impair venous return to the heart and lead to increased cerebral venous pressure with a potential increase in cerebral blood volume (CBV). On the other hand, increased intra-thoracic pressure may impair cerebral blood supply by a reduction of cardiac output. Impairment of cerebral perfusion might have negative consequences on neurological outcome. Recently, a trend toward a higher incidence of intraventricular hemorrhage (IVH) in preterm infants treated with SLI was reported [14].

As the brain is the most vulnerable organ system of the infant, data regarding side effects of SLI on cerebral hemodynamics during neonatal resuscitation are of great interest. Near-infrared spectroscopy (NIRS) may be used as a non-invasive access to the brain for evaluating cerebral oxygenation and hemodynamic changes. It has been shown in preterm infants with need for respiratory support that NIRS technology is capable of reflecting not only cerebral oxygenation but also cerebral perfusion during postnatal transition [15].

The aim of the present study was to investigate, whether SLI and subsequent PEEP treatment affected the physiological changes of CBV and improved cerebral oxygenation in infants with requirement for respiratory support by using NIRS.

## Materials and Methods

### Ethics Statement

The Regional Committee on Biomedical Research Ethics of the Medical University of Graz approved the study protocol (No. 23–302 ex 10/11).

### Trial Design

This randomized controlled pilot study was conducted at the neonatal intensive care unit (NICU) of the Medical University of Graz (Austria, Europe). Between April 2012 and December 2013 preterm infants were included into the study, if written informed consent was obtained from parents prior to birth. Follow-up data were collected until June 2014. This trial was registered at the German Clinical Trials Register (DRKS00005161) in July 2013. We are sorry to state, that we became aware of the necessity of registration during recruitment. We approve the fact, that all future trials will be registered before start. The Universal Trial Number (UTN) is U1111-1145-8147. Reporting of the study conforms to Consolidated Standards of Reporting Trials CONSORT 2010 statement [16]. CONSORT checklist and the protocol for this trial is available as supporting information; see S1 CONSORT Checklist and S1 Protocol.

### Participants

Preterm infants of gestation 28 weeks 0 days to 33 weeks 6 days delivered by elective caesarean section were included, if insufficient breathing efforts [heart rate (HR) <100 beats per minute (bpm) or irregular breathing] and/or pronounced signs of respiratory distress (grunting, tachypnea and increased work of breathing) were present. Exclusion criteria were presence of major congenital malformations, inherited disorders of metabolism and necessity of primary intubation within the first 15 minutes after birth. Criteria for endotracheal intubation were dissatisfying response to non-invasive ventilation defined as absent or insufficient increase in HR and/or preductal oxygen saturation (SpO_2_) despite supplemental oxygen. In cases of multiple births only one of the infants could be included due to technical reasons.

### Sample Size

For this randomized controlled pilot study a sample size of 40 infants was arbitrary designated and authorized by the local ethics committee. Sample size calculations were not performed according to Thabane et al. [17], since no data from previous studies were available, and this pilot study was conducted to generate data for the sample size calculation of the consecutive main study.

### Randomization

Based on a computer-generated randomization (www.randomizer.at) the included preterm infants were randomly assigned to one of the two treatment groups (SLI or control group) in 1:1 ratio. We used blocked randomization with a block size of 8. Sealed envelopes were used to conceal the randomization assignments.

### Resuscitation Procedure

**Fig 1:**
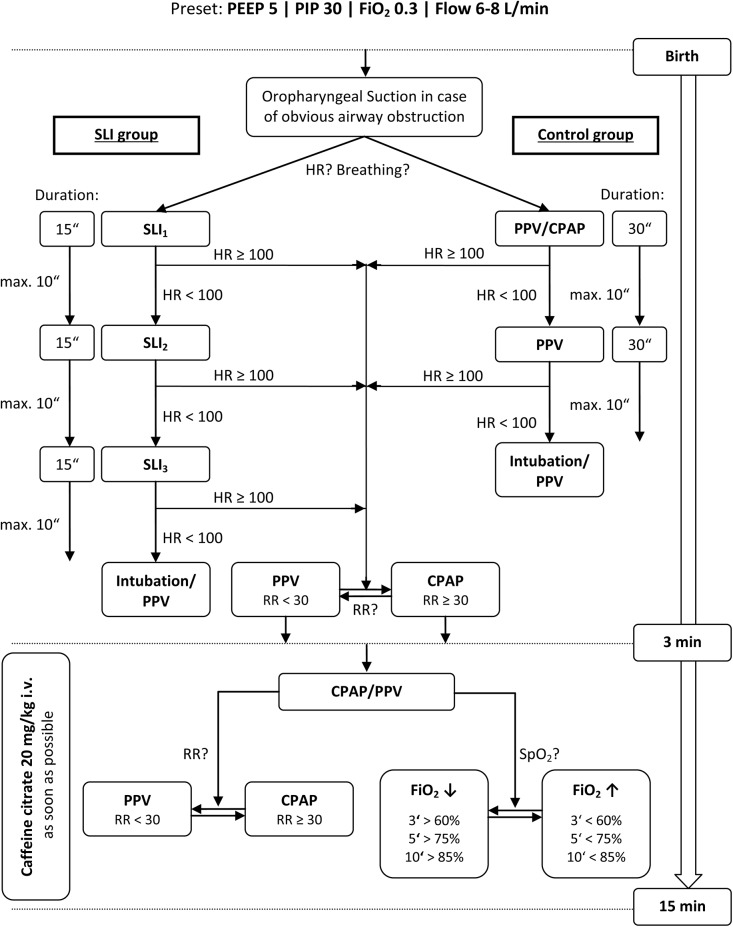
Flow chart of respiratory support in SLI and control group. After initial oropharyngeal suctioning in case of obvious airway obstruction, respiratory support was started immediately. In SLI group (left) 1 to 3 SLI maneuvers were applied, whereas in control group (right) CPAP and/or PPV was initiated. The upper part of the flow chart shows the different approaches of initial respiratory support of the two groups within the first 3 minutes after birth and the lower part shows the interventions from minute 4 to 15 after birth, which were identical in both groups. *CPAP*, continuous positive airway pressure; *FiO*_*2*_, fraction of inspired oxygen; *HR*, heart rate (beats per minute); *PEEP*, positive end exspiratory pressure (cmH_2_O); *PIP*, peak inspiratory pressure (cmH_2_O)*; PPV*, positive pressure ventilation; *RR*, respiratory rate (breaths per minute); *SLI*, sustained lung inflations.

All resuscitations of preterm infants were performed by a neonatologist or neonatal fellow. In all infants cord clamping was done within 30 seconds after delivery. Immediately after cord clamping, newborn infants were placed under an overhead heater by the midwife. If obstructions of the upper airways were obvious, immediate suction of the oropharynx was performed. In preterm infants that fulfilled the inclusion criteria respiratory support was initiated according to the study protocol (Fig 1).

In *SLI group* a SLI maneuver with duration of 15 seconds was applied via face mask at 30 cmH_2_O peak inspiratory pressure (PIP). In case of HR remaining below 100 bpm, a second and third SLI maneuver was applicable. Infants with HR > 100 bpm were supported by positive pressure ventilation (PPV) at 30 cmH_2_O PIP or CPAP at a PEEP level of 5 cmH_2_O depending on respiratory rate.

In *control group* respiratory support was provided according to recent guidelines [18]. CPAP (5 cmH_2_O PEEP) was applied in infants with respiratory rate >30 breaths per minute and signs of respiratory distress (grunting, tachypnea and increased work of breathing). Insufficient breathing efforts (HR <100 bpm, respiratory rate <30 breaths per minute or irregular breathing) indicated PPV at 30 cmH_2_O PIP via face mask.

Additionally SpO_2_ and HR were continuously monitored by a pulse oximeter fixed on the right wrist. A NIRS transducer was positioned on the newborn’s right forehead, and fixed with gauze bandage by a scientific staff member without disturbing routine medical care.

The initial fraction of inspired oxygen (FiO_2_) of 0.3 was adapted to achieve defined oxygen saturation targets (3‘: > 60%; 5‘: > 75%; 10‘: > 85%) in accordance with the 10^th^ percentile for preterm infants at 32 to 36 gestational weeks [19].

As soon as a venous line was established, a bolus of caffeine citrate (20 mg/kg body weight) was administered.

A single blood pressure measurement on the left upper arm was done 10 minutes after birth and rectal body temperature was measured 15 minutes after birth. A capillary blood sample was obtained from a heel puncture for analyzing pH, carbon dioxide partial pressure (pCO_2_), base excess, lactate and hemoglobin levels 15 minutes after birth.

During the entire study period infants were lying in supine position.

## Materials

Immediately after birth initial medical care was performed in a ‘Giraffe’ incubator (GE Healthcare; United Kingdom) or on a resuscitation desk (‘CosyCot’, Fisher&Paykel Healthcare; New Zealand). Respiratory support was applied by using a ‘Neopuff Infant T- Piece Resuscitator’ (Perivent, Fisher& Paykel Healthcare; New Zealand) and a round face mask of appropriate size (LSR Silicon mask no. 0/0 or 0/1, Laerdal; Norway). NIRS measurements were carried out with a ‘NIRO 200-NX’ tissue oxygenation monitor (Hamamatsu; Japan). Vital signs (HR, SpO_2_, blood pressure and rectal body temperature) were recorded by using an ‘IntelliVue MP30/X2 Monitor’ (Philips; The Netherlands). A ‘Florian Neonatal Respiratory Function Monitor’ (Acutronic Medical Systems; Switzerland) captured ventilation variables: tidal volume (VT), face mask leak, FiO_2_. For later analysis all parameters and the video recordings were stored using a multichannel system ‘alpha-trace digital MM’ (BEST Medical Systems; Austria).

### Near-infrared Spectroscopy

NIRS is a continuous non-invasive method to measure changes in the concentration of oxygenated (ΔHbO_2_) and reduced hemoglobin (ΔHbR). Changes in total hemoglobin (ΔHbT) can be calculated by using the following equation:

ΔHbT(μmol/l)=ΔHbO2+ΔHbR (1)

ΔHbT (µmol/l) may be converted to changes in cerebral blood volume (ΔCBV) by using a previously described relationship [20]. ΔCBV (ml/100 g brain) is calculated by the following equation, in which Hb represents the large-vessel hemoglobin concentration (g/dl):

$$\Delta CBV\;\left(\text{ml}/100\;\text{g brain}\right)\;=\Delta HbT\;*\frac{0.89}{Hb}$$ (2)

This method does not allow measurements of absolute CBV, but allows changes in CBV in proportion to HbT concentration, if Hb and the large vessel to cerebral hematocrit ratio remain constant. We presume that abrupt changes in Hb during our short-term observation were not likely to occur, since serious bleedings were not observed.

Moreover by using NIRS technology the cerebral tissue oxygenation index (cTOI) was evaluated:

$$cTOI\;\left(\%\right)=\frac{\Delta HbO2}{\Delta HbT}$$ (3)

Thus cTOI is equivalent to the cerebral regional tissue oxygen saturation (rSO_2_) of NIRS devices of other companies. NIRS measurements were conducted over the whole study period of 15 minutes using a sample rate of 2 Hz. The interoptode distance was 4 cm, and a differential path length factor of 3.85 was chosen [21].

### Outcomes

The prespecified primary outcome measures were changes in CBV and cTOI during immediate postnatal transition. The secondary outcomes were SpO_2_, HR, VT, face mask leak and FiO_2_ within the first 15 minutes after birth.

### Follow up

Each neonate was followed until discharge or until term date, depending on what came first. All-cause mortality and morbidities were recorded. Cerebral ultrasound was performed at day 1, 4, 8 and 14 after birth and at term age or before discharge, depending on what came first. The cerebral ultrasound was performed by neonatologists and pictures were reevaluated by a neonatologist (BU) blinded to the patients.

### Data Collection and Analysis

Baseline characteristics are given as mean ± standard deviation (SD) or median and range for continuous variables and absolute and relative counts for discrete variables. Group differences in baseline characteristics were analyzed using χ² and Fisher's exact tests for discrete variables, and t-test or Mann-Whitney U test for continuous variables. To analyze the courses of ΔCBV, cTOI, SpO_2_ and HR two different analyses were performed. For the first analysis values for each minute within the first 15 minutes after birth were used. For the second analysis values for every 5 seconds within the first minute after initiation of respiratory support were used. Initiation of respiratory support was defined as the time point when the mask was applied to the face for the first time. ΔCBV values for these analysis (ΔCBV_15_ and ΔCBV_0:60_) were calculated in two steps. First ΔHbT values were converted to ΔCBV as described above and second the difference of each value to the last observed value (15^th^ minute after birth for ΔCBV_15_ and 56–60^th^ second after initiation of respiratory support for ΔCBV_0:60_) was calculated. The 60 second and the 15 minute values were used as reference values, because at that time point the NIRS signal had the best quality. Courses of VT, face mask leak and FiO_2_ were calculated for each minute within the first 15 minutes only, because analyzing 5 second time intervals seemed to be inappropriate for these ventilation variables. The changes in ΔCBV_15_, cTOI, SpO_2_, HR, VT, face mask leak and FiO_2_ within the first 15 minutes after birth and ΔCBV_0:60_, cTOI, SpO_2_ and HR within the first 60 seconds after initiation of respiratory support were analyzed using a linear mixed model with fixed effects for time and group, respectively. A first order autoregressive covariance structure was used. Post hoc analysis for changes between every 5 seconds within the first minute after initiation of respiratory support, between each minute within the first 15 minutes after birth and between groups for each time point was performed. The results of the estimated model are given as mean and 95% CI. A p-value <0.05 was considered statistical significant. The statistical analyses were performed using IBM SPSS Statistics 22.0.0 (IBM Corporation; Armonk, USA).

## Results

**Fig 2:**
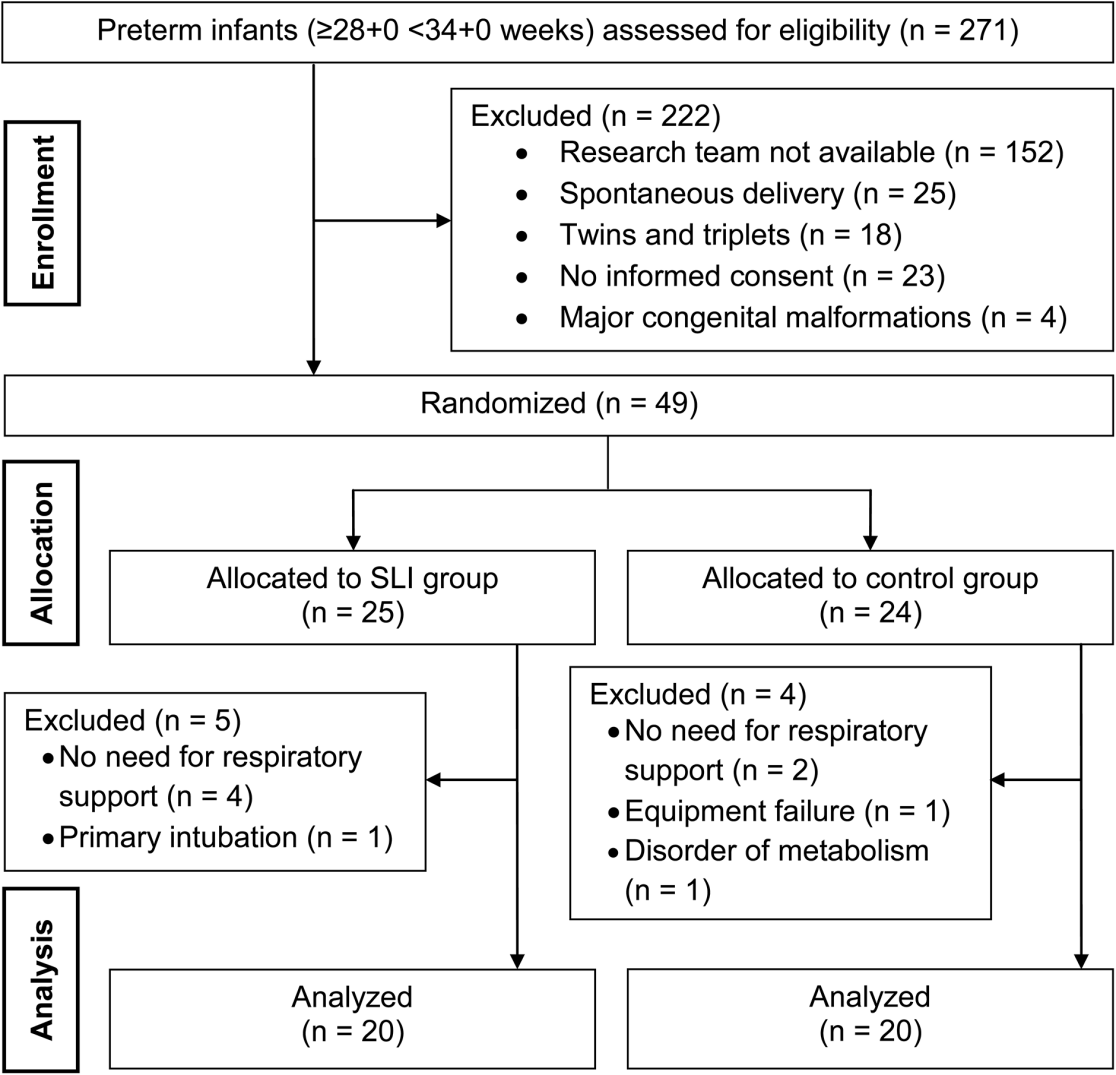
CONSORT diagram including enrollment, allocation and data analysis. *SLI*, sustained lung inflations.

271 preterm infants of gestation 28 weeks 0 days to 33 weeks 6 days were delivered in our centre between April 2012 and December 2013 and assessed for eligibility. 222 infants needed to be excluded for several reasons, which are shown in Fig 2. Therefore, 49 infants were enrolled into the study and randomly assigned to SLI or control group. A number of 7 patients did not receive the allocated respiratory support because of absence of respiratory distress or necessity of primary intubation. Another 2 infants needed to be excluded; one due to an inherited disorder of metabolism and the other one due to equipment failure. Finally, SLI and control group consisted of 20 infants each (Fig 2). The 40 included preterm infants (23 female) had a mean gestational age of 32 weeks one day (±2 days) and birth weight of 1707 (±470) g. Demographic and clinical data of the infants are summarized in Table 1 showing no significant differences between groups.

**Table 1:** Demographic and neonatal characteristics of SLI and control group. *MABP*, mean arterial blood pressure; *pCO2*, carbon dioxide partial pressure; *RS*, respiratory support.

	SLI group (n = 20)	control group (n = 20)	p-value
**Gestational age (wk),** mean (±SD)	32.1 (1.4)	32.1 (1.6)	0.94
**Gestational weight (g),** mean (±SD)	1692 (297)	1722 (604)	0.84
**Head circumference (cm),** mean (±SD)	29.7 (2.0)	29.1 (2.3)	0.35
**Female sex,** n (%)	12 (60)	11 (55)	0.75
**Multiples (twins, triplets),** n (%)	9 (45)	5 (25)	0.18
**Apgar at 1 min,** median (range)	8 (5–9)	8 (6–9)	0.58
**Apgar at 5 min,** median (range)	9 (8–9)	9 (7–10)	0.72
**Apgar at 10 min,** median (range)	9 (8–10)	9 (8–10)	1.00
**pH Umbilical artery,** mean (±SD)	7.30 (0.05)	7.31 (0.05)	0.86
**MABP (mmHg) at 10 min,** mean (±SD)	38 (9)	37 (10)	0.65
**Rectal body temperature (°C) at 15 min,** mean (±SD)	36.9 (0.5)	36.8 (0.6)	0.79
**pH at 15 min,** mean (±SD)	7.20 (0.11)	7.20 (0.08)	0.83
**pCO**_**2**_ **(mmHg) at 15 min,** mean (±SD)	64.1 (16.2)	61.8 (13.1)	0.65
**Base excess (mmol/l) at 15 min,** mean (±SD)	-3.9 (2.3)	-3.9 (2.2)	0.95
**Lactate (mmol/l) at 15 min,** mean (±SD)	3.1 (1.2)	3.1 (1.3)	0.90
**Hemoglobin (g/dl) at 15 min,** mean (±SD)	19.2 (2.4)	19.9 (1.8)	0.38
**Time of initiation of RS (s after birth),** mean (±SD)	73 (19)	75 (20)	0.72
**Caffeine citrate dosage (mg) within 15 min,** mean (±SD)	33.8 (6.6)	33.9 (10.8)	0.96

### First 15 minutes after birth

**Fig 3:**
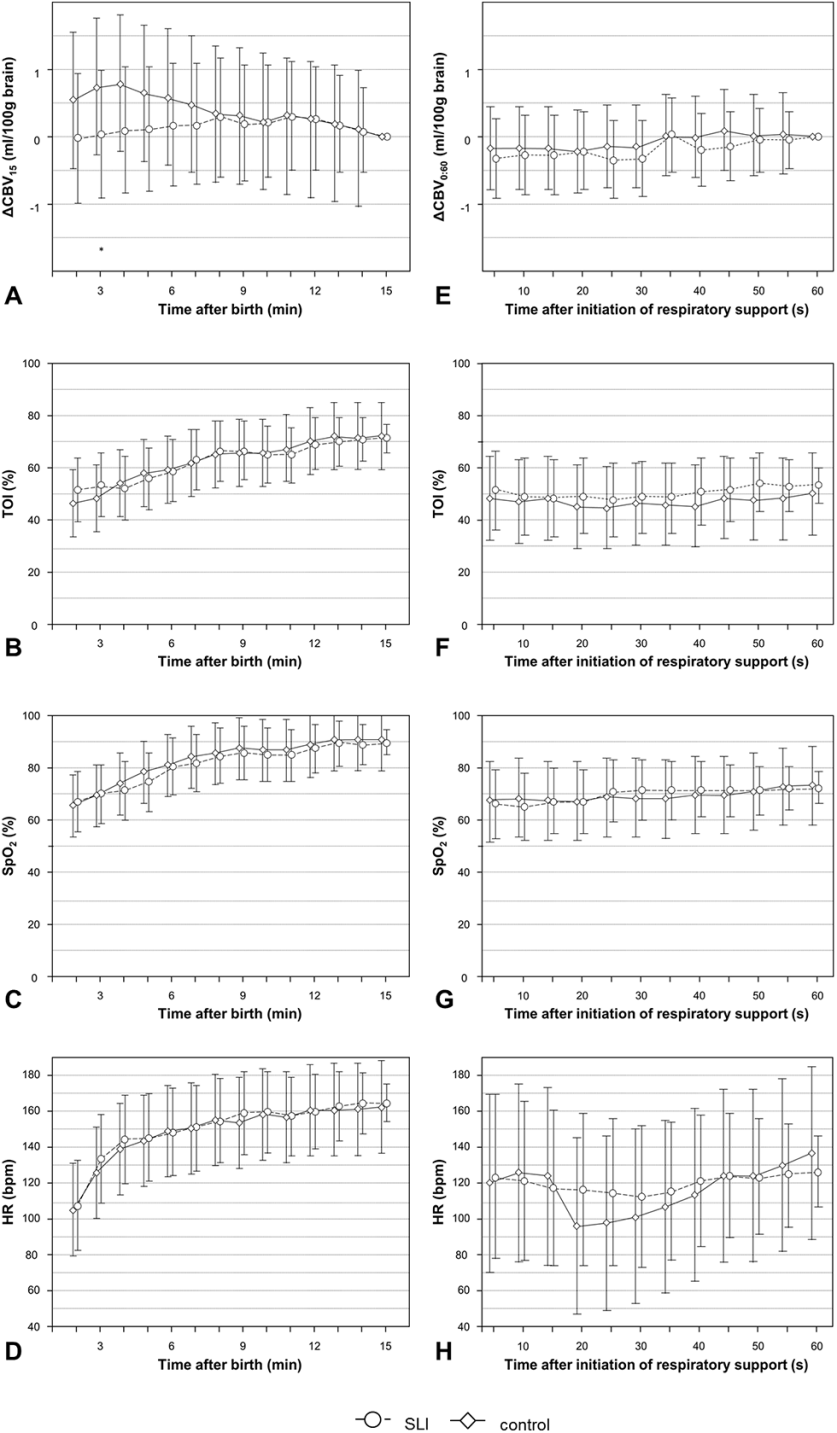
Courses of ΔCBV (A, E), cTOI (B, F), SpO_2_ (C, G) and HR (D, H) within the first 15 minutes after birth (A, B, C, D) and during the first 60 seconds after initiation of respiratory support (E, F, G, H) in SLI (circles) and control group (diamonds); values are mean (95% CI); *p < 0.05; significances are calculated between SLI and control group; ΔCBV are related to a reference value at 15 min (A) and 60 seconds (E). *CBV*, cerebral blood volume; *cTOI*, cerebral tissue oxygenation index; *HR*, heart rate; *SpO*_*2*_, peripheral arterial oxygen saturation.

ΔCBV_15_ showed a tendency toward different overall courses in both groups (p = 0.051). This was mainly caused by significantly higher ΔCBV_15_ values at minute 3 to 6 in control group compared to minute 15 [mean difference: 0.6 ml/100g (95% CI: -0.1–1.6) to 0.8 ml/100g (95% CI: -0.2–1.8)]. This represents a significant decrease in CBV in control group, whereas in SLI group ΔCBV_15_ showed similar values during the whole period of 15 minutes (Fig 3A). The higher ΔCBV_15_ values in control group led to a significant difference between groups at minute 3 (p = 0.049). Within the first 15 minutes after birth cTOI (p < 0.001), SpO_2_ (p < 0.001) and HR (p < 0.001) were significantly increasing without significant differences between both groups (Fig 3B–3D).

VT in relation to body weight was significantly increasing within the first 3 min after birth in both groups (p = 0.001) [minute 2: SLI group 4.2 ml/kg (95% CI: 0.4–8.1); control group 5.7 ml/kg (95% CI: 1.8–9.5); group difference p = 0.241; minute 3: SLI group 6.5 ml/kg (95% CI: 2.7–10.3); control group: 7.3 ml/kg (95% CI: 3.4–11.1); group difference: p = 0.517]. In the further course VT remained unchanged on a level of approximately 7 ml/kg in both groups. No significant differences between groups were observed during the entire observation period.

Face mask leak was decreasing over time (p < 0.001) in both groups. In SLI vs. control group face mask leak was 53% (95% CI: 11–95) vs. 39% (95% CI:0–81) at 5 min (group difference: p = 0.219), 28% (95% CI: 0–69) vs. 24% (95% CI: 0–66) at 10 min (group difference: p = 0.722) and 16% (95% CI: 0–34) vs. 14% (95% CI: 0–58) at 15 min (group difference: p = 0.872) without significant differences between groups during the entire observation period.

FiO_2_ did not show significant changes during the observation period (p = 0.336). In SLI vs. control group FiO_2_ was 0.32 (95% CI: 0.22–0.42) vs. 0.28 (95% CI: 0.17–0.40) at 5 min (group difference: p = 0.385), 0.27 (95% CI: 0.18–0.36) vs. 0.26 (95% CI: 0.14–0.37) at 10 min (group difference: p = 0.757) and 0.31 (95% CI: 0.26–0.36) vs. 0.27 (95% CI: 0.15–0.38) at 15 min (group difference: p = 0.191) without significant differences between groups during the entire observation period.

### First minute after initiation of respiratory support

ΔCBV_0:60_, cTOI, and SpO_2_ did not change significantly within the first 60 seconds after initiation of respiratory support in SLI and control group, nor were there any significant differences between the two groups (Fig 3E–3G). Moreover there was no significant difference between both groups in regard to HR. Nevertheless, in control group we observed a significant change in HR over time. In the control group a significant decrease in HR could be observed from 15 seconds to 30 seconds (1–5 sec: 120 bpm, 95% CI 70–170 bpm; 15–30 sec: 95 bpm, 95% CI 46–145 bpm to 101 bpm, 95% CI 52–150 bpm), while in the SLI group HR did not change significantly over time (Fig 3H).

### Blood Gas Analyses

Capillary blood samples were evaluated for pH, pCO_2_, base excess, lactate and hemoglobin levels (Table 1). There were no significant differences between groups.

### Morbidity and Respiratory Outcome

Outcome data regarding respiratory short-term outcome, mortality and morbidity at the time of hospital discharge are shown in Table 2. There were no significant differences between groups.

**Table 2:** Outcome data of SLI and control group. *BPD*, bronchopulmonary dysplasia; *FiO*_*2*_, fraction of inspired oxygen; *IVH*, intraventricular hemorrhage; *NCPAP*, nasal continuous positive airway pressure; *NEC*, necrotizing enterocolitis; *PIE*, pulmonary interstitial emphysema; *PTX*, pneumothorax; *PVL*, periventricular leukomalacia; *ROP*, retinopathy of the prematurity; *SLI*, sustained lung inflations.

	SLI group (n = 20)	control group (n = 20)	p-value
**Intubation within the first 24 hours of life,** n (%)	7 (35)	5 (25)	0.49
**Mechanical ventilation at 24 hours of life,** n (%)	3 (15)	5 (25)	0.69
**NCPAP at 24 hours of life,** n (%)	3 (15)	5 (25)	0.69
**FiO**_**2**_ **at 24 hours of life,** median (range)	0.21 (0.21–0.21)	0.21 (0.21–0.40)	0.43
**Need for Surfactant,** n (%)	6 (30)	6 (30)	1
**BPD,** n (%)	0 (0)	1 (5)	1
**PTX (drainage),** n (%)	0 (0)	1 (5)	1
**PIE,** n (%)	1 (5)	0 (0)	1
**IVH I + II°,** n (%)	1 (5)	1 (5)	1
**IVH III + IV°,** n (%)	0 (0)	0 (0)	-
**PVL,** n (%)	2 (10)	0 (0)	0.49
**ROP,** n (%)	0 (0)	0 (0)	-
**NEC,** n (%)	0 (0)	0 (0)	-
**Mortality,** n (%)	0 (0)	0 (0)	-
**Duration of hospitalization (d),** median (range)	26.5 (15–71)	26 (14–116)	0.93

## Discussion

To the best of our knowledge this randomized controlled trial is the first study that incorporated detailed analysis of CBV during postnatal transition in preterm infants receiving respiratory support. The intervention group was treated by applying SLI maneuvers at birth and compared to control group, which received respiratory standard care. CBV behavior was significantly different in infants with SLI compared to control group. Whereas CBV showed a significant decrease from minutes 3 to 15 in control group, CBV basically remained unchanged in SLI group.

### Cerebral hemodynamics

CBV is dependent on cerebral arterial blood supply and on venous drainage. Mean airway pressure possibly affects both. Cerebral arterial blood supply is dependent on cardiac output and vascular resistance. Cardiac output depends on HR and stroke volume. Whereas there were differences in the course of HR within the first minute after initiation of respiratory support, overall no significant differences between groups were observed. Stroke volume is largely determined by venous return (Frank-Starling law) [22]. An increase in airway pressure potentially results in a decrease in venous return, thus decreasing preload [23, 24]. Therefore, the increased intrathoracic pressure during SLI might have reduced preload, resulting in impairment of stroke volume. Moreover, a direct compressive effect of high airway pressure on the newborn heart was described [23, 25]. Unfortunately we cannot provide information about changes in cardiac output in our study population. However, a decrease in cardiac output in response to high airway pressure during SLI does not conflict with our findings of unaltered CBV in SLI group. First, in case of reduced cardiac output cerebral blood supply might not be affected due to a decrease in cerebral vascular resistance in infants with adequate cerebral autoregulatory capacity. Second, the effects of high pulmonary vascular resistance caused by increased mean airway pressure during SLI on the systemic arterial circulation might also be minimized due to right to left shunting via foramen ovale and ductus arteriosus within the first minutes of life [26]. Therefore, we hypothesize that the reason for the differences in CBV behavior was not caused primarily by altered arterial blood supply, but by an impairment of cerebral venous return.

Recently, a significant decrease in CBV within the first 15 minutes of life in healthy term infants was demonstrated by our study group [27]. We hypothesized that changes in blood gases during this time period are the main cause for our observation. Compared to these findings, CBV behavior was different in the present study population (preterm infants with need for respiratory support). In control group the CBV decrease was less pronounced compared to healthy term infants, in SLI group CBV did not change at all within the study period.

In preterm infants it has already been demonstrated that increased airway pressure was associated with a decrease in superior vena cava blood flow [28]. The increased mean airway pressure during SLI might have led to impaired cerebral venous return and consecutively to cerebral venous stasis, resulting in absence of CBV decrease.

It remains unclear, whether respiratory support itself or the condition of the infant leading to requirement for respiratory support is responsible for the differences in CBV behavior compared to healthy term infants. Nevertheless, impaired cerebral venous return may predispose to IVH [29] or periventricular hemorrhagic infarction [30]. A trend toward a higher incidence of IVH in preterm infants after SLI treatment was reported [14].

### Cerebral tissue oxygenation

cTOI was significantly increasing within the study period without any differences between both groups. The increase in regional oxygen saturation was according to previous publications (30–33). Reference ranges for cTOI in preterm infants measured by NIRO ‘200-NX’ are currently not available. Recently, our study group published reference ranges of cerebral tissue oxygenation for term infants without need for respiratory support by using an ‘INVOS 5100C’ device (Covidien, USA) [31]. Moreover we published data of cerebral tissue oxygenation in preterm infants with and without respiratory support by using the same device [32]. By using a ‘Foresight’ device (CAS Medical Systems, USA), Fuchs et al. (2012) published percentiles for cerebral tissue oxygenation of very low birth weight infants during neonatal resuscitation, in whom SLI were applied [33]. Values of cerebral tissue oxygenation measured with different NIRS devices have to be interpreted with caution, although comparative studies with all the widely used devices have already been published [34]. In addition, the comparability of our findings to these studies is limited due to differences in gestational age and requirement of respiratory support.

### Pulmonary effects of SLI

SLI is supposed to assist lung aeration thus supporting the establishment of FRC, which might improve oxygenation [9, 10]. Recently, it has been shown in preterm infants that a SLI of 10 seconds and 25 cmH_2_O at birth was not effective unless the infants breathed. As FRC gain was only associated with spontaneous breathing, active glottic adduction was addressed to be responsible for most SLI failures [35]. To determine the effectiveness of SLI in our study population, various variables including oxygenation parameters were examined. We particularly looked into the courses of SpO_2_ and cTOI, but did not find significant differences between both groups during observation time. Thus, in our study SLI was not capable to improve oxygenation. Moreover we didn’t find differences between groups regarding respiratory function (VT, face mask leak, FiO_2_), blood gases at 15 minutes and respiratory short-term outcome. Whereas we couldn’t find evidence for pulmonary improvement after applying SLI, we demonstrated an impact on cerebral hemodynamics. Therefore, further investigations on SLI at birth should not only focus on respiratory outcome but also on the consequences on the developing brain.

### Limitations

Our study has several limitations. First, all preterm infants were delivered by caesarean section and immediate cord clamping was performed. We have no information, whether vaginal birth or delayed cord clamping would have resulted in a different behavior of CBV and cTOI in our study population. Our study group already described differences in cerebral tissue oxygenation in term infants during immediate transition after elective cesarean and vaginal delivery [36].

We decided to use a NIRO 200-NX for this study because it was the only available NIRS monitor which supported CBV measurements. The inclusion criteria regarding the gestational age was determined at 28 weeks 0 days to 33 weeks 6 days. The exclusion of extremely low birth weight (ELBW) infants was necessary, because at the time of study initiation NIRS probes of Hamamatsu (Japan) were pretty bulky and not suitable for measurements on the smallest of our preterm infants. Therefore, our data do not permit conclusions about CBV behavior in ELBW infants.

It has been shown that cerebral vasoreactivity is dependent on pO_2_ and pCO_2_ levels in human newborn infants [20, 37]. Taking this into account, we continuously observed SpO_2_ which is closely linked to pO_2_ on the one hand and obtained capillary blood gases for pCO_2_ evaluation at one time point (15 minutes postnatal) on the other hand. Unfortunately, we cannot provide continuous data for pCO_2_, which might be beneficial for the interpretation of CBV results.

In addition, no data were collected on the impact of respiratory support on cardiac output and the superior vena cava flow indicating the extent of venous return from the brain.

## Conclusion

This is the first study that demonstrated an impact of SLI on cerebral hemodynamics. In contrast to the CBV decrease in control group, CBV remained unchanged in SLI group during the observation period. We hypothesize that after SLI an impaired venous return to the heart leads to cerebral venous stasis and the potential hazardous differences in CBV behavior. Until now, studies on respiratory interventions only focusing on improvement in respiratory outcome might have unanticipated negative consequences on the brain. Thus further studies are warranted including reconfirmation of the present findings in infants with lower gestational age. Future SLI studies should include cerebral perfusion within outcome parameters, or at least neurodevelopmental outcome at two years of age.

## Supporting Information

S1 CONSORT Checklist CONSORT 2010 checklist of information to include when reporting a randomized trial. (PDF)

S1 Protocol Trial study protocol. (PDF)
