# Supplementary material for: Do Sustained Lung Inflations during Neonatal Resuscitation Affect Cerebral Blood Volume in Preterm Infants? A Randomized Controlled Pilot Study
Source: PLoS One. 2015 Sep 25;10(9):e0138964. doi: 10.1371/journal.pone.0138964 (PMC4583511; doi:10.1371/journal.pone.0138964)
Supplement: S1 Protocol — (PDF) [file pone.0138964.s002.pdf]

Pilot Study: Does Sustained Lung Inflation at Birth Improve Cerebral Oxygen Saturation in Preterm Infants ?

Berndt Urlesberger, MD

Department of Pediatrics, Division of Neonatology, Medical University of Graz, Auenbruggerplatz 30, 8036 Graz, Austria

Keywords: preterm infant, transition, sustained lung inflation, near infrared spectroscopy, arterial oxygen saturation, regional cerebral oxygen saturation, cerebral blood volume

Address for correspondence:

Univ. Prof. Dr. Berndt Urlesberger

Klinische Abeilung für Neonatologie

Univ. Klinik für Kinder- und Jugendheilkunde

Medizinische Universität Graz

Auenbruggerplatz 30

8036 Graz

Phone: +43-316-385-81133

Fax: +43-316-385-2678

Mail: berndt.urlesberger@medunigraz.at

1. Abstract

Transition from fetus to newborn is a complex process with an ongoing discussion about the use of supplemental oxygen. It is unknown which oxygen concentration is appropriate. As the brain is the most vulnerable organ system of the infant, a direct way to assess its oxygenation would be potentially useful. Such a new approach is the measurement of regional cerebral oxygen saturation using nearinfrared spectroscopy.

Many preterm infants need positive pressure ventilation during the transition to air breathing at birth to assist lung aeration and the initiation of pulmonary gas ex-change. It is possible that an initial sustained inflation (SI) supports the transitional period of the lungs. Clinical studies have shown lower intubation rates when a SI is given. There are no data of cerebral side effects of SI.

The aim of the present study is to investigate in preterm infants with need of respiratory support, whether a SI immediately after birth improves cerebral oxygen saturation.

2. Background

2.1. Near Infrared Spectroscopy

Near-infrared-spectroscopy (NIRS) enables non-invasive measurement of oxygenation in regions of interest e.g. cerebral, renal and "peripheral muscle" tissue. In 1977 the principle was described by Jöbsis[i]. NIRS is based on two fundamental facts: the relative transparency of biological tissue to near infrared light (wavelength 700-1000nm) and the presence of chromophores (colour bearing compounds) in the biological tissue, which have oxygenation-dependent absorption properties (e.g. haemoglobin and cytochrome oxidase) in the near infrared region. NIRS enables therefore the noninvasive continuous measurement (of changes in the concentration) of oxygenated haemoglobin (HbO2), deoxygenated haemoglobin (Hb) and cytochrome oxidase (CytOx).

Furthermore, spatially resolved spectroscopy, a new NIRS method, was recently introduced for evaluation of regional tissue oxygen saturation (rSO2)[ii]. With this technology it is possible to measure regional tissue oxygen saturation in different organ systems (brain, kidney, liver, muscle, and others) or body regions (pre-, post-ductal peripheral tissue).

Regional tissue oxygen saturation depends on the local balance of oxygen delivery and oxygen consumption, and the regional arterial/venous volume ratio, reflecting oxygen saturation in veins (70-80%), capillaries (5%) and arteries (15-25%)[iii]. The main component

of tissue oxygen saturation is venous blood, thus representing oxygen saturation after oxygen consumption by the tissue. Therefore, the values for regional oxygen saturation tend to be close to venous oxygen saturation, but do not equate venous saturation[3].

2.2 NIRS and changes in cerebral haemodynamics and oxygenation

Numerous studies investigated cerebral haemodynamics during different interventions[iv]:
Effects of suctioning on cerebral haemodynamics during ventilation were studied and as expected there was a fall cerebral oxygenation[v]. The effect of surfactant administration on cerebral blood volume (CBV) was studied. Whereas Hb and HbO2 changes seemed to be according to changes of arterial oxygen saturation, CBV showed inconsistent changes within different studies. Using NIRS, administration of aminophyllin and indomethacin showed significant effects on cerebral haemodynamics, whereas caffeine and ibuprofen had no or only small effects[vi],[vii].
Further studies investigated the effects of different clinical situations and procedures such as a patent ductus arteriosus[viii], blood pressure changes[ix], cord clamping[x], and blood sampling from umbilical artery catheter[xi] on cerebral haemodynamics.
Our group investigated changes of cerebral oxygenation during periodic breathing[xii], and during episodes of apnoea[xiii,xiv,xv] in preterm infants. Furthermore, we used tilting manoeuvres to investigate the cerebral autoregulative capacity, showing that lower gestational age was associated with increased changes of CBV[xvi], and that preterm infants with periventricular leucencephalomalacia showed reduced cerebral autoregulative capacity[xvii]. In these studies the NIRO 500 and 300 (Hamamtsu, Japan) were used.

2.3 Transition of the newborn and oxygen saturation

Transition from fetus to newborn is a complex physiological process. Within the recent years interest has grown in the use of pulsoximetry to monitor arterial oxygen saturation during this transitional period[xviii,xix,xx,xxi]. All newborn infants have oxygen desaturation at birth. A newborn undergoing normal postnatal transition needs more than 5 minutes to attain an arterial oxygen saturation >80% and almost 10 minutes to reach 90%. Several studies have shown, that there are significant differences in the course of arterial oxygen saturation (SpO2) according to the mode of delivery. Newborn infants after elective CS showed lower SpO2 values during transition compared to infants after vaginal delivery [14-17].
There is an ongoing discussion about the use of supplemental oxygen during neonatal

resuscitation, because it is unknown which oxygen concentration is appropriate for preterm and term infants during resuscitation. As the brain is the most vulnerable organ system of the infant, a more direct way to assess its oxygenation in a simple non-invasive way would be potentially useful. A new approach to cerebral oxygenation is the measurement of regional oxygen saturation using NIRS. There are some reports of regional oxygen saturation of the brain on the first day of life [xxii], during the first weeks [xxiii,xxiv,xxv,xxvi], and during transition phase [xxvii,xxviii].

Our group published data on cerebral oxygen saturation within the first week of life[xxix], and for the first time in literature in a large group of newborn infants during transition[xxx]. We have shown, that during transition of the newborn dynamics of changes in regional cerebral oxygen saturation (rSO2brain) and pulsoximetric arterial oxygen saturation (SpO2) were not paralleling each other. rSO2brain did not show changes in periods were SpO2 significantly changed. Therefore, measurement of both, arterial and regional oxygen saturation is necessary to estimate cerebral oxygen supply. In these studies the Invos™ Cerebral/Somatic Oximeter monitor (Covidien, USA) was used.

2.4 Sustained lung inflation

Many preterm infants need positive pressure ventilation (PPV) during the transition to air breathing at birth to assist lung aeration and the initiation of pulmonary gas ex-change. It is possible that an initial sustained inflation (SI) might overcome the long time constant of a liquid-filled lung and facilitate early gas exchange to reduce the need for intubation[xxxi,xxxii]. Clinical studies have shown lower intubation rates when a SI is given[xxxiii,xxxiv]. However, this procedure is not commonly used because of the risk of overinflating and damaging the lung during the initial inflation[xxxv]. Despite this, the international guidelines on neonatal resuscitation state that an initial SI may be beneficial during stabilization at birth[xxxvi].

In animal experiments with preterm rabbits it has ben shown, that duration of SI has to be between 10 and 20 seconds to fully aerate the lung and to establish an adequate functional residual capacity (FRC)[xxxvii]. Using this duration there were no signs of overinflation of the premature lung whatsoever. Furthermore it was shown, that SI plus subsequent application of positive endexspiratory pressure (PEEP) using a CPAP device promoted uniform lung aeration and created a much larger FRC at birth than ventilation without these techniques[xxxviii]. Very recently it has been shown that in preterm infants with respiratory distress syndrome, SI may decrease the need for mechanical ventilation without inducing evident adverse effects[xxxix]. Until now there is no literature, showing whether a SI after birth

results in different SpO2 values, and thus a different need for supplemental oxygen during resuscitation.

3. Aims of the present study

The aim of the present study is to investigate in preterm infants with need of respiratory support, whether a SI immediately after birth influences cerebral oxygen saturation and cerebral blood volume. There are three specific aims.

3.1 Hypothesis I: SI and SpO2

According to physiology an increase in FRC with improvement of lung aeration may result in increase of SpO2, and thus a reduction of supplemental oxygen. The first hypothesis is, that use of SI increases SpO2 values, and reduces the need of supplememtal oxygen.

3.2 Hypothesis II: SI and rSO2brain

According to physiology an increase of SpO2 may result in an increase of rSO2brain. The second hypothesis is, that the use of SI increases rSO2brain values.

3.3 Hypothesis III: SI and cerebral blood volume

SI has not shown any serious side effects in clinical trials untill now, but it may be associated with an significant change in CBV. Any positive pressure put on the thoracic wall may increase cerebral venous pressure, and may have influence on heart rate and cardiac output. Therefore, it remains unclear, whether the SI procedure impairs cerebral blood supply. It is unclear, whether there is reduction of cardiac output with reduction of CBV, or whether the increase on cerebral venous pressure results in increase of CBV. The third hypothesis is, that the use of SI significantly changes CBV.

4. Patients and Methods

4.1 Patients

Preterm neonates with a gestational age $\geq$28 and <34 weeks born at the Department of Obstetrics and Gynaecology, Medical University of Graz, who need respiratory support

during transition after birth (done by a neonatologist of the Department of Pediatrics, Medical University of Graz), will be enrolled into the study.
Neonates with congenital malformations and neonates, who need primary intubation will be excluded.

4.2 Methods

4.2.1 Study design

Prospective randomised observational study.

4.2.2 Protocol

The medical history of any pathologic findings during pregnancy and birth will be collected. Gestational age, birth weight, circumference and diameter of arm and calf, gender, pH of umbilical artery and APGAR score will be documented in each neonate.
Resuscitation will be performed according the "European Consensus Guidelines" on the management of neonatal respiratory distress syndrome[xl] and the guidelines for neonatal resuscitation of the American Heart Association (of 2010)[xli]. CPAP with mask will be performed with Neopuff Infant Resuscitator (Fisher&Paykel Healthcare, NewZealand). This T-piece ventilator is a pressure-limited mechanical device that supplies consistent peak inspiratory pressure (PIP) and PEEP, and is capable of delivering sustained inflation (SI). To avoid pressure leakage, a neonatal mask of appropriate size is used, which adequately coveres both the mouth and nostrils of infants. To be sure about mask leakage the non-invasive respiratory-function monitoring is used.

4.2.3 Monitoring

The following non-invasive monitoring will be combined immediately after birth for the first 15 minutes of life:

1) Pulse oximetry and heart rate (IntelliVue MP50, Philips, Netherland)
2) Blood pressure measurement (IntelliVue MP50, Philips, Netherland)
3) Central temperature (IntelliVue MP50, Philips, Netherland)
4) NIRS (NIRO 200, Hamamatsu, Japan))
5) Video camera
6) Respiratory function monitor (Florian respirator monitors, Acutronic Medical Systems, Switzerland)

Data of the different devices will be stored in a polygraphic system (alpha-trace digitalMM, B.E.S.T. Medical Systems, Austria) for further analysis.

4.2.3.1 Pulse oximetry – arterial oxygen saturation and heart rate

For pulsoximetry the IntelliVue MP50 monitor (Philips, Netherland) will be used. The pulse oximetry sensor will be applied on the right palm or wrist immediately after birth for monitoring preductal arterial oxygen saturation and heart rate.

4.2.3.2 Blood pressure

For non-invasive blood pressure measurements the IntelliVue MP50 monitor (Philips, Netherland) will be used. The pneumatic cuff will be placed around the left upper arm and blood pressure measurements will be performed five and ten minutes after birth.

4.2.3.3 Central temperature

For noninvasive temperature measurements the IntelliVue MP50 monitor (Philips, Netherland) will be used. A rectal sensor will be placed for central temperature measurement.

4.2.3.4 NIRS

For NIRS measurements the NIRO 200 (Hamamatsu, Japan) will be used. This monitor uses a "continuous wave spatially resolved" technique for measurement of regional oxygen saturation (rSO2), this parameter is called tissue oxygenation index (TOI) within this device. Furthermore this monitor uses continuous wave technology to measure the following parameters: oxygenated haemoglobin (HbO2) and deoxygenated haemoglobin (Hb). The sum of these two is calculated and expressed as total haemoglobin (Hbtot). Changes of Hbtot represent changes in cerebral blood volume (CBV).
At the moment, the NIRO 200 is the only device, that is technically able to measure both, changes in regional cerebral oxygen saturation and changes in CBV, and is easy enough to use to be able to place a sensor immediately after birth, and to measure during the transition of the neonate. Very often the Invos™ Cerebral/Somatic Oximeter monitor (Covidien, USA) is used during transition, but this device will not give any information on changes in CBV.
Immediately after birth a cerebral sensor will be placed on the left forehead.

4.2.3.5 Video camera

A video camera will record the term and preterm neonates during the whole measurement/transition time (first 15 minutes of life). ***The camera is positioned in such a way, that no person can be identified (only hands can be seen). The recorded sequences allow identification of artefacts, and secure correct data analysis during sustained inflation.***

4.2.3.6. Respiratory function monitoring during CPAP or PPV with face mask

For respiratory function monitoring the Florian respirator monitor (Acutronic Medical Systems, Switzerland) will be used. It measures airway pressure and gas flow in and out of a face mask with a hot wire anemometer and its successful application during resuscitation has already been shown (57,58).

During CPAP or PPV the Florian respirator monitor displays continuously:

1. positive inspiratory pressure
2. positive end expiratory pressure
3. inspiratory and expiratory flow
4. inspiratory and expiratory tidal volume
5. leak between mask and face
6. respiratory rate
7. minute volume
8. spontaneous breathing

4.2.3 Randomisation

Preterm infants will be randomly assigned to one of two groups:

Group A: Standard resuscitation with CPAP (5cmH2O PEEP) and PPV (PIP 30cmH2O) with Neopuff Infant Resuscitator

Group B: Start with SI (PIP 30cmH2O) with duration of 15 sec, after that standard resuscitation with CPAP (5cmH2O PEEP) and PPV (PIP 30cmH2O) with Neopuff Infant Resuscitator. Only if heart rate continues to be below 100/min SI may be repeated twice.

The randomization happens via internet using the "Randomizer", a software program provided by the Institute for Medical Informatics, Statistics and Information, Medical University Graz.

There will only be descriptive statistical analysis.

4.2.4 Standard resuscitation protocol

Ensure that the heart rate is >100bpm at minute 1.

If breathing is insufficient (none or insufficient breathing movements, retractions and nasal flaring) CPAP with mask will be applied with Neopuff Infant Resuscitator. CPAP is used with a PEEP of 5cm H2O, and intermittent positive pressure ventilation is used with a PIP of 30cmH2O (standard settings).

The goal of oxygen saturation should be an oxygen saturation value in the interquartile range of preductal saturations measured in healthy term babies following vaginal birth at sea level (minute 3: 70-75%, minute 5: 80-85%, minute 10: 85-95%)[37]. These targets may be achieved by initiating resuscitation with FiO2 of 0.3 and titrating the oxygen concentration to achieve an SpO2 in the target range as described above using pulse oximetry.

5. Research plan and time table

The project may start in Sept 2011 and is designed for a two year running time. Start with pilot study to ensure calculation of power (40 infants, 20 in each group).

[i] *Jöbsis FF. Noninvasive infrared monitoring of cerebral and myocardial oxygen sufficiency and circulatory parameters. Sience 1977; 198:1264–1267.*

[ii] *Suzuki S, Takasaki S, Ozaki T, Kobayashi Y. A tissue oxygenation monitor using NIR spatially resolved spectroscopy. Proc SPIE. 1999; 3597:582–592*

[iii] *H M Watzman, C D Kurth, L M Montenegro, J Rome, J M Steven, S C Nicolson. Arterial and venous contributions to near-infrared cerebral oximetry. Anesthesiology 2000; 93:947-53*

[iv] *Greisen G. Is near-infrared spectroscopy living up to its promises? Semin Fetal Neonatal Med 2006; 11:498-502*

[v] *Effect of surfactant administration on cerebral haemodynamics and oxygenation in premature infants--a near infrared spectroscopy study. Roll C, Knief J, Horsch S, Hanssler L. Neuropediatrics. 2000 Feb;31(1):16-23.*

[vi] *Nicklin SE, Hassan IA, Wickramasinghe YA, Spencer SA. The light still shines, but not that brightly? The current status of perinatal near infrared spectroscopy. Arch Dis Child Fetal Neonatal Ed 2003; 88(4):F263–F28.*

[vii] *Wolfberg AJ, du Plessis AJ. Near-infrared spectroscopy in the fetus and neonate. Clin Perinatol. 2006;33(3):707-728.*

[viii] *Lemmers PM, Toet MC, van Bel F. Impact of patent ductus arteriosus and subsequent therapy with indomethacin on cerebral oxygenation in preterm infants. Pediatrics. 2008 Jan;121(1):142-147.*

[ix] *Suresh V, Marson AG, Appleton RE, Beirne M, Weindling AM. Relationship between blood pressure, cerebral electrical activity, cerebral fractional oxygen extraction, and peripheral blood flow in very low birth weight newborn infants. Pediatr Res 2006; 59(2):314–319.*

[x] *Baenziger O, Stolkin F, Keel M, von Siebenthal K, Fauchere JC, Das Kundu S, Dietz V, Bucher HU, Wolf M. The influence of the timing of cord clamping on postnatal cerebral oxygenation in preterm neonates: a randomized, controlled trial. Pediatrics. 2007 Mar;119(3):455-459.*

[xi] *Roll C, Hüning B, Käunicke M, Krug J, Horsch S. Umbilical artery catheter blood sampling volume and velocity: impact on cerebral blood volume and oxygenation in very-low-birthweight infants. Acta Paediatr. 2006 Jan;95(1):68-73.*

[xii] *Urlesberger B, Pichler G, Gradnitzer E, Reiterer F, Zobel G, Muller W. Changes in cerebral blood volume and cerebral oxygenation during periodic breathing in term infants. Neuropediatrics 2000;31:75-81*

[xiii] *Pichler G, Schmölzer G, Müller W, Urlesberger B. Body position dependent changes in cerebral hemodynamics during apnea in preterm infants. Brain Dev 2001;23:395-400.*

[xiv] *Pichler G, Urlesberger B, Müller W. Impact of bradycardia on cerebral oxygenation and cerebral blood volume during apnoea in preterm infants. Physiological Measurements 2003; 24: 671-680.*

[xv] *Urlesberger B, Kaspirek A, Pichler G, Müller W. Apnoea of prematurity and changes in cerebral oxygenation and cerebral blood volume. Neuropediatrics 1999;30:208-211*

[xvi] *Pichler G, van Boetzelar MC, Müller W, Urlesberger B. Effect of tilting on cerebral blood volume in preterm and term infants. Biol Neonate 2001; 80: 179-185*

[xvii] *Pichler G, Urlesberger B, Schmölzer G, Müller W. Effect of tilting on cerebral haemodynamics in preterm infants with periventricular leucencephalomalacia. Acta Paediatr 2004; 93(1): 70-5*

[xviii] *Kamlin CO, O'Donnell CP, Davis PG, Morley CJ. Oxygen saturation in healthy infants immediately after birth. J Pediatr 2006; 148:585–589*

[xix] *Rabi Y, Yee W, Chen SY, Singhal N. Oxygen saturation trends immediately after birth. J Pediatr 2006; 148:590 –594*

[xx] *Finer N, Leoni T. Oxygen Saturation monitoring for the preterm infant: the evidence basis for the current practice. Pediatr Res 2009; 65:375-80*

[xxi] *J A Dawson, P G Davis, C P F O'Donnell, C O F Kamlin, C J Morley. Pulse oximetry for monitoring infants in the delivery room: a review. Arch Dis Child Fetal Neonatal Ed 2007; 92:F4-7*

[xxii] *Sorensen LC, Greisen G. The brains of very preterm newborns in clinically stable condition may be hyperoxygenated. Pediatrics 2009; 124:958-63.*

[xxiii] *E A Verhagen, H J ter Horst, P Keating, A F Bos. The course of cerebral oxygen saturation and oxygen extraction during the first two weeks of life in pre term infants. Acta Pædiatrica 2007; 96 (Suppl. 456):140*

[xxiv] *Naulaers G, Morren G, Van Huffel S, Casaer P, Devlieger H. Cerebral tissue oxygenation index in very premature infants. Arch Dis Child Fetal Neonatal Ed. 2002; 87: F189 –F192*

[xxv] *Naulaers G, Morren G, Van Huffel S, Casaer P, Devlieger H. Measurement of tissue oxygenation index during the first three days in premature born infants. Adv Exp Med Biol. 2003; 510:379 –383*

[xxvi] *van den Berg, E, Lemmers, P M A, Toet, M C, Klaessens, J H G, van Bel, F. Effect of the "InSurE" procedure on cerebral oxygenation and electrical brain activity of the preterm infant. Arch Dis Child Fetal Neonatal Ed 2010; 95:F53-8*

[xxvii] *K Isobe, T Kusaka, Y Fujikawa, K Okubo, K Nagano, S Yasuda, M Kondo, S Itoh, K Hirao, S Onishi. Measurement of cerebral oxygenation in neonates after vaginal delivery and cesarean section using full-spectrum near infrared spectroscopy. Comp Biochem Physiol, Part A Mol Integr Physiol. 2002; 132:133-138*

[xxviii] *Fauchere JC, Schulz G, Haensse D, Keller E, Ersch J, Bucher HU, Wolf M. Near-Infrared spectroscopy measurements of cerebral oxygenation in newborns during immediate portnatal adaptation. J Pediatrics 2009; epub ahead press*

[xxix] *Grossauer K, Pichler G, Schmölzer G, Zotter H, Mueller W, Urlesberger B. Comparison of peripheral and cerebral tissue oxygenation index in neonates. Arch Dis Child Fetal Neonatal Ed 2009; 94:F156*

[xxx] *Urlesberger B, Grossauer K, Pocivalnik M, Avian A, Müller W, Pichler G. Regional oxygen saturation of the brain and peripheral tissue during birth transition of term infants. J Pediatr 2010,157:740-4*

[xxxi] *Hird MF, Greenough A, Gamsu HR. Inflating pressures for effective resuscitation of preterm infants. Early Hum Dev 1991, 26:69 –72*

[xxxii] *Vyas H, Milner AD, Hopkin IE, Boon AW. Physiologic responses to prolonged and slow-rise inflation in the resuscitation of the asphyxiated newborn infant. J Pediatr 1981, 99:635– 639*

[xxxiii] *Lindner W, Hogel J, Pohlandt F Sustained pressure-controlled inflation or intermittent mandatory ventilation in preterm infants in the delivery room? A randomized, controlled trial on initial respiratory support via nasopharyngeal tube. Acta Paediatr2005, 94:303–309*

[xxxiv] *te Pas AB, Walther FJ A randomized, controlled trial of delivery-room respiratory management in very preterm infants. Pediatrics 2007 120:322–329*

[xxxv] *Bjorklund LJ, Ingimarsson J, Curstedt T, John J, Robertson B, Werner O, Vilstrup CT Manual ventilation with a few large breaths at birth compromises the therapeutic effect of subsequent surfactant replacement in immature lambs. PediatrRes 1997 42:348 –355*

[xxxvi] *The International Liaison Committee on Resuscitation The International Liaison Committee on Resuscitation (ILCOR) consensus on science with treatment recommendations for pediatric and neonatal patients: neonatal resuscitation. Pediatrics 2006 117:e978 – e988*

[xxxvii] *Te Pas AB, Siew M, Wallace MJ, Kitchen MJ, Fouras A, Lewis RA, Yagi N, Uesugi K, Donath S, Davis PG, Morley CJ, Hooper SB. Effect of sustained inflation length on establishing functional residual capacity at birth in ventilated premature rabbits. Pediatr Res2009 66:295-300*

[xxxviii] *Te Pas AB, Siew M, Wallace MJ, Kitchen MJ, Fouras A, Lewis RA, Yagi N, Uesugi K, Donath S, Davis PG, Morley CJ, Hooper SB. Establishing functional residual capacity at birth: the effect of sustained inflation and positive end-expiratory pressure in a preterm rabbit model. Pediatr Res 2009 65:537-41*

[xxxix] *Lista G, Fontana P, Castoldi F, Cavigioli F, Dani C. Does Sustained Lung Inflation at Birth Improve Outcome of Preterm Infants at Risk for Respiratory Distress Syndrome? Neonatology 2011 99:45–50*

[xl] *Sweet D, Bevilacqua G, Carnielli V, Greisen G, Plavka R, Didrik Saugstad O, Simeoni U, Speer CP, Valls-I-Soler A, Halliday H, Working Group on Prematurity of the World Association of Perinatal Medicine , European Association of Perinatal Medicine . European consensus guidelines on the management of neonatal respiratory distress syndrome. J Perinat Med. 2007;35(3):175-186.*

[xli] *John Kattwinkel, Jeffrey M. Perlman, et al. Neonatal Resuscitation: 2010 American Heart Association Guidelines for Cardiopulmonary Resuscitation and Emergency Cardiovascular Care. Circulation 2010;122;S909-S919*
